# In vitro evaluation of the antimicrobial activity of chlorhexidine alone or in combination with ketoconazole or miconazole against clinical isolates of Malassezia pachydermatis and multidrug-resistant Staphylococcus pseudintermedius

**DOI:** 10.1099/mic.0.001701

**Published:** 2026-04-28

**Authors:** Gianna Goldman, Domenico Santoro

**Affiliations:** 1 Department of Small Animal Clinical Sciences, University of Florida, Gainesville, FL, USA

**Keywords:** azoles, chlorhexidine, *Malassezia pachydermatis*, multidrug resistance, *Staphylococcus pseudintermedius*

## Abstract

**Background.** *Staphylococcus pseudintermedius* (*SP*) and *Malassezia pachydermatis* (*MP*) are common causes of canine skin infections associated with increasing antimicrobial resistance. Topical products containing chlorhexidine and azoles have been utilized to treat these infections. However, a limited number of studies have assessed the interactions between chlorhexidine and azoles against *SP* and *MP*.

**Hypothesis/Objectives.** To assess efficacy and potential additivity/synergy between chlorhexidine and azoles against clinical isolates of *MP* and multidrug-resistant *SP* (*MDR-SP*).

**Materials and Methods.** A total of 30 *MDR-SP* and 30 *MP* isolates were tested using a modified broth microdilution method. Six twofold dilutions of 2% chlorhexidine gluconate, 1% miconazole nitrate and 0.15% ketoconazole were tested alone and in combination. Minimum inhibitory (MIC) and bactericidal/fungicidal (MBC/MFC) concentrations were recorded, and synergy, additivity or antagonism was calculated.

**Result.** For *MDR-SP*, the combinations of chlorhexidine/miconazole (*P*=0.003) and chlorhexidine/ketoconazole (*P*<0.0001) resulted in lower MICs than chlorhexidine alone. Only the chlorhexidine/ketoconazole combination had lower MBCs (*P*=0.0071) than chlorhexidine alone. For *MP*, only the chlorhexidine/ketoconazole combination had lower MICs (*P*<0.0001) than chlorhexidine alone. Both combinations of chlorhexidine/miconazole (*P*=0.0028) and chlorhexidine/ketoconazole (*P*<0.0001) resulted in lower MFCs than chlorhexidine alone. Chlorhexidine/miconazole combination showed synergy in three *MDR-SP* isolates, but in none of the *MP* isolates. Chlorhexidine/ketoconazole showed synergy for 1 out of 30 *MDR-SP* and 19 out of 30 *MP* isolates.

**Conclusions and Clinical Relevance.** These results suggest that chlorhexidine/azole combinations are an effective topical treatment for *MDR-SP* and MP infections. Further studies should assess the efficacy of commercial products containing these compounds and their efficacy *in vivo*.

**Impact Statement**

Both animal and human medicine are battling with antimicrobial resistance. Therefore, there is an urgent need to develop novel alternative therapeutic options that are effective, safe, affordable and less prone to driving resistance. In this scenario, topical medications containing chlorhexidine and azoles are commonly used. However, no studies have shown a beneficial effect of adding azoles to chlorhexidine to treat bacteria and/or yeast infections. This study shows how the association between chlorhexidine and azoles (miconazole or ketoconazole) does have synergistic or additive effects decreasing the concentration of chlorhexidine needed to inhibit and kill staphylococcal bacteria and *Malassezia* organisms.

## Introduction

Organisms belonging to the genera *Staphylococcus* and *Malassezia* are common residents of normal cutaneous and mucosa microbiota in both animals and humans. *Staphylococcus pseudintermedius* (*SP*) is the most common species associated with canine skin infection, one of the most common infections observed in dogs. On the other hand, *Malassezia pachydermatis* (*MP*) is the most frequently isolated yeast from the skin and ears in dogs [1]. Both organisms have been associated with significant antimicrobial resistance, especially exacerbated by continuous and sometimes indiscriminate use of systemic antimicrobials [2,4]. The prevalence of multidrug-resistant *SP* (*MDR-SP*) isolates, with an acquired resistance of at least one antimicrobial in three or more drug classes [5], has significantly increased over the past decade [2,3]. The prevalence of multidrug resistance varies across geographic locations, with methicillin resistance increasing the odds for an isolate to be multidrug resistant [2]. There have also been increasing reports of antifungal resistance in clinical isolates of *MP*, with some studies reporting as high as 30% of clinical isolates resistant to azoles like ketoconazole [4,6, 7].

With antimicrobial resistance rising with the use of systemic antimicrobials, there has been a significant push to utilize topical antimicrobials. Most recent guidelines on the treatment of superficial pyoderma suggest that these infections should be treated only with topical antimicrobials, such as chlorhexidine [8,9]. Chlorhexidine digluconate is a topical antiseptic and antimicrobial agent that can be used against both Gram-positive and Gram-negative organisms, targeting and binding to cell membranes and leading to a leakage of cell components [10,11]. *In vitro* resistance to chlorhexidine has been seen in *S. aureus*, but little to no resistance has been detected in *SP* [10]. In veterinary patients, chlorhexidine has been widely used across concentrations ranging from 0.5% to 4% in forms such as sprays, wipes, shampoos and mousses. Many of these commercial chlorhexidine products also contain azole antifungals, such as miconazole and ketoconazole.

Miconazole has previously demonstrated anti-staphylococcal activity [12,13]. When miconazole is combined with chlorhexidine, it demonstrates synergy in about 30% of isolates of *SP* [14,15]. Ketoconazole’s efficacy against *SP* and *MP* has never been tested alone or in combination with chlorhexidine. Ketoconazole, like miconazole, is commonly found within chlorhexidine topicals, but the effect of this pairing on the most common causes of canine pyoderma is unknown. Thus, the objective of this study was to assess the efficacy and potential additive and/or synergy between chlorhexidine and ketoconazole or miconazole against clinical isolates of MDR-*SP* and *MP*.

## Methods

### Isolates

Thirty clinical isolates of MDR-*SP* and thirty clinical isolates of *MP* were used for this study. All the clinical isolates were collected from the skin and/or ears of allergic (MDR-*SP* and *MP*) as well as non-allergic dogs (*MP*). The isolates were previously collected by the Clinical Microbiology Service at the authors’ institution and subsequently banked in glycerol beads. Each isolate was identified based on colony morphology, biochemical features and via MALDI-TOF MS with an Autoflex system (Bruker, Billerica, MA, USA) using the directed protocol according to the manufacturer’s instructions. The Trek Sensititre (Trek Diagnostic Systems, Inc.; Cleveland, OH, USA) automated system, according to the Clinical and Laboratory Standards Institute guidelines [16], was used to determine the antimicrobial susceptibility for the *MDR-SP* isolates. The MDR status of each bacterial organism was verified based on susceptibility results and based on published guidelines to identify MDR organisms [5].

### Isolate preparation

Each assay was quality-controlled with a corresponding American Type Culture Collection (ATCC) strain: ATCC 49444 *SP* and ATCC 14522 *MP* (Table S1, available in the online Supplementary Material). All bacterial samples were subcultured on Columbia Agar Plates with 5% sheep blood (Hardy Diagnostics; Santa Maria, CA, USA) at 37 °C for 24 h in a room air incubator. All yeast samples were subcultured onto Sabouraud dextrose agar (Hardy Diagnostics) at 37 °C for 72 h in a room air incubator. Colonies were then inoculated into sterile water until a 0.5 McFarland Standard was achieved using the Den-1B McFarland Densitometer (Grant Instruments; Shepreth, Cambridgeshire, UK). Then, for both organisms, the inoculum was diluted into Sensititre^™^ cation-adjusted Mueller–Hinton broth (MHB) with TES broth (Remel, San Diego, CA, USA) to reach a concentration of 1×10^6^ c.f.u. ml^−1^.

### Treatment preparations

Treatment formulations included pharmaceutical-grade 2% chlorhexidine gluconate (Aspen Veterinary Resources Ltd., Liberty, MO, USA), 1% miconazole nitrate (Miconosol lotion 1% – Phoenix Pharmaceuticals, Inc.; San Joseph, MO, USA) and 2% ketoconazole cream (Fougera Pharmaceuticals Inc.; Melville, NY, USA). The 2% ketoconazole cream was diluted down to a 0.15% solution with 100% DMSO (Valhoma Corporation, Tulsa, OK, USA). Treatments were diluted with sterile PBS, then into MHB (Remel) to working concentrations.

### MIC assay

A broth microdilution methodology was utilized to determine the MIC of both *MDR-SP* and *MP* [17]. The concentration ranges for the treatments were chosen based on previous MIC data in chlorhexidine [18] and miconazole [14,15], as well as multiple trials utilizing ATCC isolates to determine ketoconazole. Six twofold dilutions of each compound were tested alone or eight twofold dilutions in combination (1 : 1 ratio) with chlorhexidine. For *MDR-SP*, chlorhexidine was tested at dilutions ranging from 12 μg ml^−1^ to 0.375 μg ml^−1^. Miconazole nitrate was tested at dilutions ranging from 8 μg ml^−1^ to 0.25 μg ml^−1^. Ketoconazole was tested at 160 μg ml^−1^ to 5 μg ml^−1^. The chlorhexidine/miconazole combination was made in eight twofold dilutions starting at 12 µg ml^−1^ of chlorhexidine and 8 µg ml^−1^ of miconazole. The chlorhexidine/ketoconazole combination was made in eight twofold dilutions starting at 12 µg ml^−1^ of chlorhexidine and 160 µg ml^−1^ of ketoconazole. The twofold serial dilutions of treatment were added to each well containing MHB (100 µl) and 100 µl of inoculum, leading to a final inoculum in each well of 5×10^5^ c.f.u. ml^−1^. Negative control wells (contamination control) contained 200 µl of MHB without inoculum, while positive control wells (growth control) contained 100 µl of MHB and 100 µl of inoculum. Each plate was incubated for 20–24 h (*MDR-SP*) or 72 h (MP) at 37 °C in a room air incubator. The ATCC strains of *SP* and *MP* were tested as experimental and internal controls. All experimental conditions were tested in duplicate to assess for technical variability. Polyethylene glycol 400 (PEG400), a typical bactericidal agent, was listed as an ingredient in the miconazole nitrate used as a treatment but, due to the amount of dilution performed, should have exhibited no effect on the bacterial isolates [19].

The MIC was identified as the lowest concentration void of a visible pellet after incubation. The MIC discrepancies between duplicate wells were allowed within one dilution. In these cases, the higher concentration was considered. This assay was also used to determine the MIC_50_ and MIC_90_ of the bacterial and fungal sample population. These were defined as the lowest concentration of an antimicrobial agent required to inhibit the growth of 50% (MIC_50_) and 90% (MIC_90_) of the bacterial or fungal isolates tested (15 out of 30 and 27 out of 30 isolates, respectively).

### Minimum bactericidal/fungicidal concentration assay

Measurement of the minimum bactericidal or fungicidal concentration (MBC/MFC) was determined by inoculating 10 µl from the MIC wells and at least three dilutions above the MIC per each treatment onto Colombia Agar Plates with 5% sheep blood (*MDR-SP*) or Sabouraud dextrose (*MP*) agar (Hardy Diagnostics) and incubated for 20–24 h (*MDR-SP*) or 72 h (MP). The MBC/MFC was reported as the highest dilution that did not permit colony growth. The MBC/MFC_50_ and MBC/MFC_90_ were defined as the lowest concentration of an antimicrobial required to kill 50% and 90% of the bacterial or fungal isolates tested (15 out of 30 and 27 out of 30 isolates, respectively).

### Modified time-kill method

A modified time-kill method was used to assess the interaction between chlorhexidine and the azoles [10,20]. The methodology used in this study is identical to the one described in [10] with the only exception that only time 0 and 24 h were assessed in this study in lieu of a full time-kill assay reported previously [20]. Briefly, at time 0 and 24 h post-incubation, 10 µl from all wells per each treatment was inoculated onto Colombia Agar Plates with 5% sheep blood (*MDR-SP*) or Sabouraud dextrose (*MP*) agar (Hardy Diagnostics) and incubated for 20–24 h (*MDR-SP*) or 72 h (MP). Then, colonies were counted, and the median colony count was represented graphically. Synergy was defined as a 2-log_10_ decrease in colony count between the combinations of treatments and chlorhexidine. Additivity or an indifference interaction was defined as a 1-log_10_ decrease in colony count between the combinations of treatments and chlorhexidine. Antagonism was defined as a 2-log_10_ increase in colony count between a combination of treatments and chlorhexidine.

### Statistical analysis

Statistical analysis was applied to the MIC and MBC/MFC data to compare the combination of chlorhexidine and azoles with chlorhexidine alone at the same concentration. The collected data were first tested for normal distribution using the Shapiro–Wilk test (*α*=0.05). The MIC and MBC/MFC data were reported as medians. A Friedman’s test followed by a Dunn’s multiple comparison test as post-hoc analysis was performed to compare MIC and MBC/MFC data of chlorhexidine alone and in combination with azoles. *P* values of ≤0.05 were considered statistically significant. All statistical analysis was performed via PRISM v10 (GraphPad Software Inc.; La Jolla, CA, USA).

## Results

### Multi-drug resistant *SP*

The median, MIC/MBC, MIC/MBC_50_ and MIC/MBC_90_ results for the chlorhexidine, miconazole and ketoconazole and their respective combinations against *MDR*-*SP* are displayed in Table 1. Chlorhexidine was the most active single agent against *MDR-SP* with a median MIC, MIC_50_ and MIC_90_ of 1.5 µg ml^−1^. Only eight (26.7%) isolates reached an MBC for miconazole at 8 µg ml^−1^; thus, an MBC_50_/MBC_90_ was unable to be determined. Ketoconazole was ineffective (lack of an MIC or MBC) against *MDR-SP* at the concentrations tested, consistently generating growth at all concentrations tested (Table 1).

**Table 1.** MIC and minimum bactericidal concentration (MBC) data for chlorhexidine, miconazole and ketoconazole and respective combinations (Chlor/Mico and Chlor/Keto) in 30 clinical isolates of *MDR-SP*. nd: not determined

	Chlorhexidine (µg/ml)	Miconazole (µg/ml)	Ketoconazole (µg/ml)	Chlor/Mico (µg/ml)	Chlor/Keto (µg/ml)
**Median MIC**	1.5	2	nd	0.75/0.5	0.75/10
**MIC_50_**	1.5	4	nd	0.75/0.5	0.75/10
**MIC_90_**	1.5	4	nd	1.5/1.0	0.75/10
**Median MBC**	3	8	nd	3.0/2.0	3.0/40
**MBC_50_**	3	nd	nd	3.0/2.0	3.0/40
**MBC_90_**	6	nd	nd	6.0/4.0	3.0/40

**Fig. 1.**
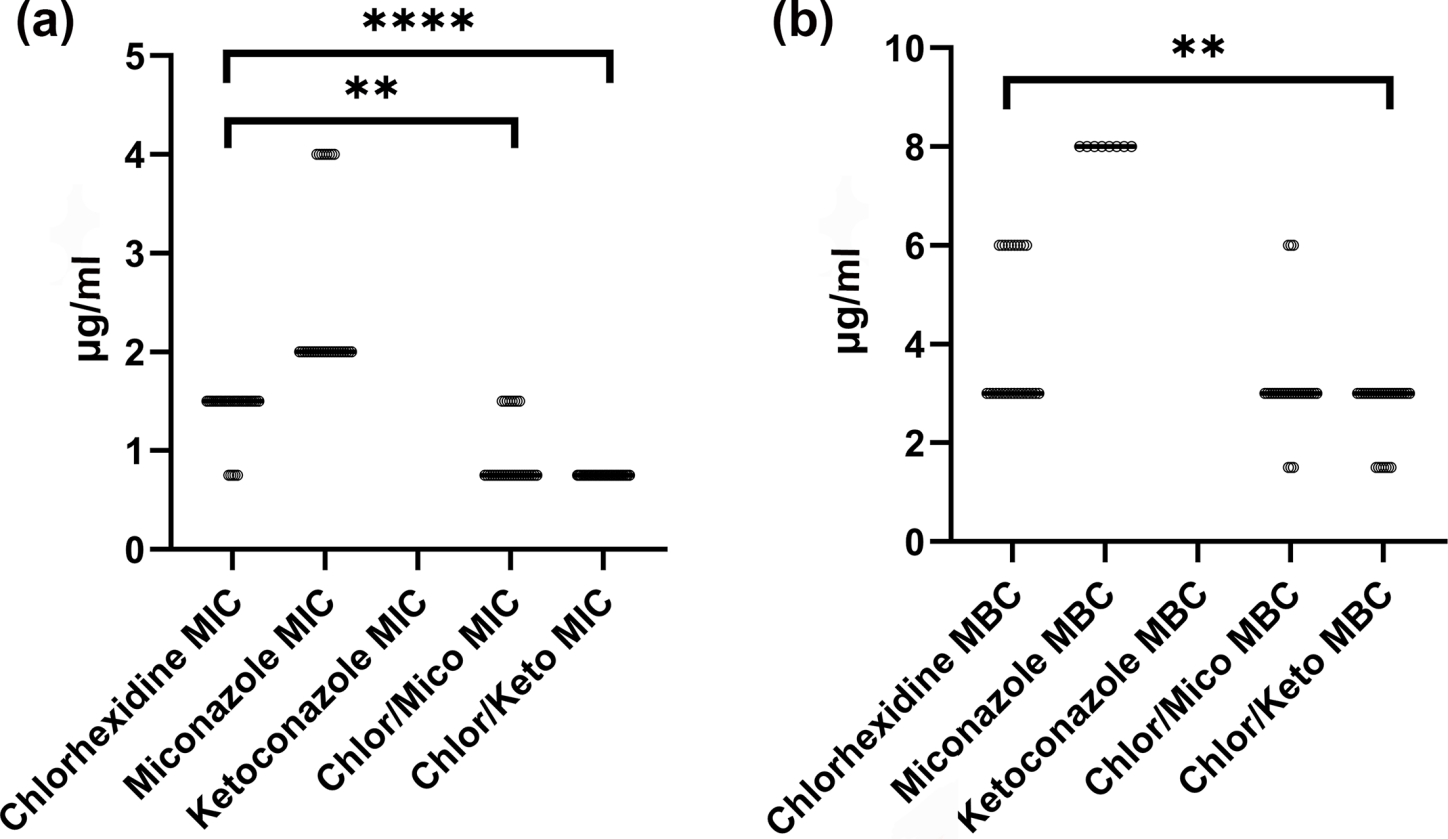
MIC (**a**) and MBC (**b**) of chlorhexidine alone or in combination with miconazole (Chlor/Mico) or ketoconazole (Chlor/Keto) for 30 clinical isolates of *MDR-SP*. Points represent MIC/MBC values for individual isolates. **P<0.01; ****P<0.0001.

The combinations of chlorhexidine and miconazole (*P=0.003*) and chlorhexidine and ketoconazole (*P<0.0001*) both significantly reduced the MIC of *MDR-SP* compared with chlorhexidine alone (Fig. 1). The addition of the azoles to chlorhexidine was able to reduce the MIC of chlorhexidine by one dilution, though the MIC_90_ of the chlorhexidine/miconazole combination remained the same as the chlorhexidine MIC_90_, while the chlorhexidine/ketoconazole combination stayed at 0.75 µg ml^−1^ for chlorhexidine and 10 µg ml^−1^ for ketoconazole. The MBC of the combination of chlorhexidine/miconazole was not significantly reduced compared to chlorhexidine alone (*P*=0.2). However, the MBC of the chlorhexidine/ketoconazole combination was one dilution lower than that of chlorhexidine alone, which was statistically significant (*P*=0.007). The chlorhexidine/miconazole combination did not reduce either the MBC_50_ (3 µg ml^−1^) or the MBC_90_ (6 µg ml^−1^) compared with the MBC_50/90_ of chlorhexidine alone (3 µg ml^−1^ and 6 µg ml^−1^, respectively). The chlorhexidine/ketoconazole combination did not reduce the MBC_50_ (3 µg ml^−1^) but did reduce the MBC_90_ (3 µg ml^−1^) compared with the MBC_50/90_ of chlorhexidine alone (3 µg ml^−1^ and 6 µg ml^−1^, respectively).

Comparing the combination of chlorhexidine/miconazole to chlorhexidine/ketoconazole, there was a lack of significant difference in activity (MIC: *P=0.36* and MBC: *P*=0.69) (Fig. 1).

### M. pachydermatis

The median MIC/MFC, MIC/MFC_50_ and MIC/MFC_90_ results for the chlorhexidine, miconazole and ketoconazole and their respective combinations against *MP* are displayed in Table 2. Ketoconazole was the most active agent against *MP* with a median MIC, an MIC_50_ and an MIC_90_ of 0.04 µg ml^−1^. The MIC largely matched the MFC in most cases. Although less effective than ketoconazole, miconazole was also effective against *MP*. Chlorhexidine was less active than the antifungals (Table 2).

**Table 2.** MIC and minimum fungicidal concentration (MFC) data for chlorhexidine, miconazole and ketoconazole and respective combinations (Chlor/Mico and Chlor/Keto) in 30 clinical isolates of *MP*

	Chlorhexidine (µg/ml)	Miconazole (µg/ml)	Ketoconazole (µg/ml)	Chlor/Mico (µg/ml)	Chlor/Keto (µg/ml)
**Median MIC**	1	0.16	0.04	1/0.08	0.5/0.02
**MIC_50_**	1	0.16	0.04	1/0.08	0.5/0.02
**MIC_90_**	2	0.32	0.04	1/0.08	1/0.04
**Median MBC**	2	0.24	0.04	1/0.08	0.5/0.02
**MBC_50_**	1	0.32	0.04	1/0.08	0.5/0.02
**MBC_90_**	4	0.64	0.08	1/0.08	1/0.04

**Fig. 2.**
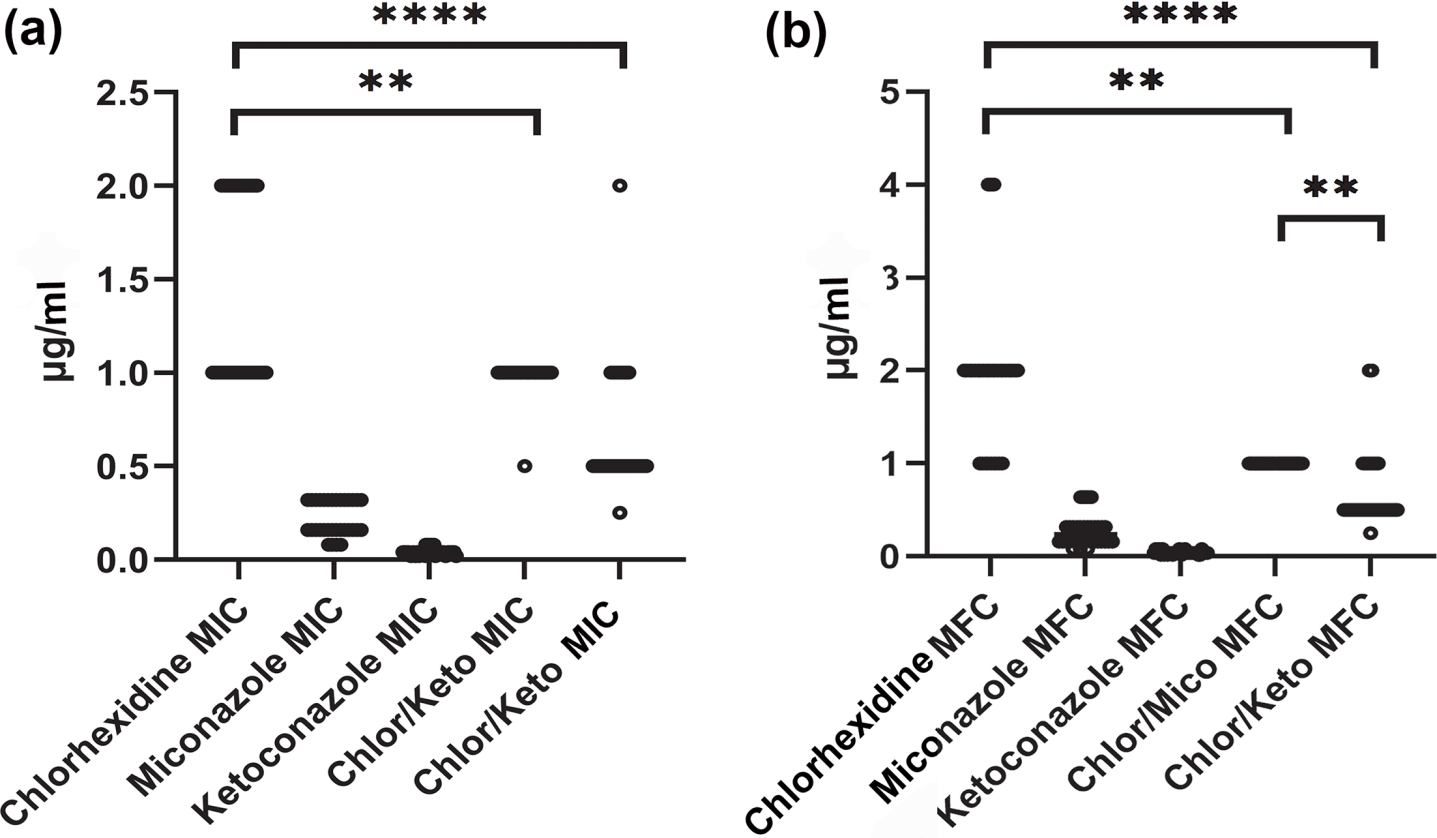
MIC (**a**) and MFC (**b**) of chlorhexidine alone or in combination with miconazole (Chlor/Mico) or ketoconazole (Chlor/Keto) for 30 clinical isolates of *MP*. Points represent MIC/MFC values for individual isolates. **P<0.01; ****P<0.0001.

The combination of chlorhexidine/miconazole did not significantly reduce the MIC against *MP* (*P=0.12*). However, the combination of chlorhexidine/ketoconazole did significantly lower the MIC (*P<0.0001*) (Fig. 2). The chlorhexidine/miconazole combination was able to significantly reduce (by one dilution) the MFC when compared to chlorhexidine alone (*P=0.0028*). The chlorhexidine/miconazole combination did not reduce the MFC_50_ (1 µg ml^−1^) but did reduce the MFC_90_ (1 µg ml^−1^) compared with the MFC_50/90_ of chlorhexidine alone (1 µg ml^−1^ and 4 µg ml^−1^, respectively). The chlorhexidine/ketoconazole combination reduced the MFC by two dilutions when compared to chlorhexidine alone (*P<0.0001*) (Fig. 2). The chlorhexidine/ketoconazole combination reduced both the MFC_50_ (0.5 µg ml^−1^) and the MFC_90_ (1 µg ml^−1^) compared with the MFC_50/90_ of chlorhexidine alone (1 µg ml^−1^ and 4 µg ml^−1^, respectively).

Comparing the combination of chlorhexidine/miconazole to chlorhexidine/ketoconazole, the latter was associated with a higher antifungal activity of chlorhexidine in both MIC (*P*=0.002) and MFC (*P=0.01*).

### Modified time-kill assay

In *MDR-SP*, a synergistic effect was noted for the chlorhexidine/miconazole combination in 3 out of 30 (10%) isolates and for the chlorhexidine/ketoconazole combination in 1 out of 30 (3%) isolates (Table S2). An additive effect was seen using the chlorhexidine/miconazole combination in 8 out of 30 (26.6%) isolates, whereas there was no effect using the chlorhexidine/ketoconazole combination for any of the isolates (Table S2). No antagonistic effects were noted in either combination (Table S2). Lack of any effect was seen for the ATCC 49444 (Table S1).

Chlorhexidine/miconazole combination did not exhibit a synergistic effect in any of the *MP* isolates, but a synergistic effect was seen with the chlorhexidine/ketoconazole combination for 19 out of 30 (59%) isolates (Table S3). A lack of additive effect was seen for the chlorhexidine/ketoconazole combination in any of the isolates, whereas an additive effect of the chlorhexidine/miconazole combination was seen in 1 out of 30 (3%) isolates (Table S3). Finally, an antagonistic effect was noted using the chlorhexidine/miconazole combination in 15 out of 30 (47%) isolates and using the chlorhexidine/ketoconazole combination in 4 out of 30 (12.5%) isolates (Table S3). Similarly, an antagonistic effect was noted using the chlorhexidine/miconazole combination in the ATCC 14522 (Table S1).

## Discussion

The results of this study confirmed that chlorhexidine alone or in combination with ketoconazole or miconazole is an effective treatment for both *MDR-SP* and *MP*. In addition, lower MICs of both *MDR-SP* and *MP* were seen when chlorhexidine was combined with an azole when compared with chlorhexidine alone. In *MDR-SP*, the combination of chlorhexidine/ketoconazole had a lower MBC than chlorhexidine alone, although it did not exhibit any significant additivity or synergy. The combination of chlorhexidine and miconazole, however, did show synergism in 10% of isolates and additivity in 30% of isolates, which is concurrent with results in previous investigations [14,15]. In *MP*, the MFC of both combinations of chlorhexidine/miconazole and chlorhexidine/ketoconazole was reduced when compared with chlorhexidine alone. A synergistic relationship was found between chlorhexidine and ketoconazole in 19 out of 30 tested *MP* isolates. An antagonistic effect was noted in 15 out of 30 isolates tested with the chlorhexidine and miconazole combination.

Azoles are known for their strong antifungal activity; however, their direct antibacterial activity, although suggested, has been only sporadically demonstrated. In the present study, ketoconazole alone did not exhibit any antibacterial activity against *MDR-SP*. Such results are in contrast with previous literature showing that ketoconazole had a minimal bacteriostatic activity against *S. aureus* [21]. However, the intrinsic differences between *S. aureus* and *SP*, as well as the possible previous exposure to azoles in the clinical isolates tested in this study, could be at the base of this discrepancy. Miconazole, on the other hand, has been shown to have activity against Gram-positive organisms, both in this and in a previous study [22]. The proposed mechanism of action of miconazole activity against *Staphylococcus* spp. is based on the inhibition of 14α-sterol-demethylase homologues in staphylococcal bacteria [22,24]. Alternatively, as more recent literature suggests, it could be associated with an inhibition of bacterial flavohaemoglobins, which play a role in bacterial resistance to nitrosative stress [22,25].

Although commonly proposed in veterinary dermatology, there has been a lack of literature investigating the synergistic effect of chlorhexidine with azoles. This is the first study showing potential synergy or additivity between chlorhexidine and azoles against few isolates of veterinary-associated pathogens. In addition, these results show a lack of difference in antibacterial effects when chlorhexidine is combined with either miconazole or ketoconazole. While the reason behind the enhanced efficacy of chlorhexidine when combined with azole antifungals is currently unknown, it could possibly be associated with the ability of chlorhexidine to disrupt the cell membrane of bacteria, allowing the azole antifungal to enter more readily into cells, or the opposite, with the azoles causing reversible disruption to the cell membrane that chlorhexidine is able to act upon. In this study, although ketoconazole was not associated with any antibacterial activity against *MDR-SP*, it was able to enhance the effectiveness of chlorhexidine. These results suggest the possibility of an underlying mechanism in which ketoconazole facilitates the penetration of chlorhexidine through the bacterial wall, improving chlorhexidine’s activity. Future studies are needed to further investigate such a theory.

As far as the antifungal activity of chlorhexidine and azoles is concerned, this is very well known. The former is the most used topical against yeast infections in animals, and the latter represents the most used class of antifungals used topically and systemically. This is the first study showing a significant enhancement of chlorhexidine antifungal activity when combined with azoles. Of particular note is the synergistic effect between chlorhexidine and ketoconazole against most of the *MP* clinical isolates tested. Interestingly, the combination of chlorhexidine/miconazole, while lowering both the MIC and MFC significantly, did not have any synergistic effect and only one additive effect when compared to chlorhexidine alone. Miconazole and chlorhexidine have traditionally been combined as a treatment against *MP*, so multiple isolates showed high colony counts despite the combination being a surprising result. Both azoles often exhibited an MIC that was equivalent to their MFC, indicating a fungicidal effect at the concentrations tested. On the contrary, miconazole had an overall bacteriostatic effect against the 30 isolates of *MDR-SP* tested, as only 8 isolates generated an MBC.

Very few studies have been performed on *Malassezia* susceptibility in general, but chlorhexidine on its own has been shown to be very effective against *Malassezia in vitro* [18]. Combinations of azoles with chlorhexidine against *MP* have not been strongly investigated previously. In an earlier clinical trial of 54 dogs comparing 3% chlorhexidine shampoo and 2% miconazole/2% chlorhexidine shampoo, it was shown that the combination shampoo did not perform any better than the 3% chlorhexidine shampoo [25,26]. On the other hand, synergistic interactions between chlorhexidine acetate and miconazole have been reported for other yeasts like *Candida albicans* [27]. The present study provided insights on how chlorhexidine and ketoconazole and miconazole combinations interact within *MP*, though further studies should assess how these combinations work *in vivo*. Finally, azole resistance and tolerance are becoming increasingly more common in yeasts due to the use of systemic antimicrobials [4]. The combination of azoles and chlorhexidine may help to expand chlorhexidine’s efficacy while limiting the use of systemic antimicrobials [12]. The use of topical antifungals and azoles resulting in tolerance and resistance is unknown but should be further investigated.

There were a few limitations in this study. First, a true time-kill assay was not performed. Synergy was still able to be evaluated based on the single time point, but a full time-kill study would be the most ideal method to assess these interactions, considering multiple concentrations of chlorhexidine and azoles. However, given that the aim of this study was to specifically assess the concentration of chlorhexidine and azoles present in commercially available products, a true gradient of concentration was outside of the scope of this series of experiments. Controls were not run for all ingredients listed in each treatment. In particular, DMSO was included in the positive and negative biological controls without affecting the viability of either organism. As for the miconazole solution, a control was not implemented for the inactive ingredients of the commercially available lotion (PEG400 and ethyl alcohol). However, after dilutions, the concentration of such ingredients was much below the antimicrobial concentrations previously reported (1 : 1250 of PEG400 at the highest miconazole concentration tested *vs*. 1 : 250 previously reported for *Staphylococcus aureus*) [28] (0.044% of ethyl alcohol at the highest miconazole concentration tested *vs*. 80–85% recommended for *S. aureus*) [29]. Finally, although the scope of the study was to assess a clinically representative sample, each isolate was only independently run once, opening for possible variation in the repeatability of the results. However, given the extreme similarities and low variation of the MICs and MBCs among the 30 clinical isolates, it is possible that minimal variation would be expected.

The reduction of MICs/MBCs/MFCs by the combination of chlorhexidine and ketoconazole compared to chlorhexidine in both *MDR-SP* and *MP* suggests that chlorhexidine/ketoconazole is an effective topical combination to treat both *SP* and *MP* infections. In addition, the chlorhexidine/ketoconazole combination, although expressing similar antibacterial activity, may be superior to the chlorhexidine/miconazole combination against *MP* isolates. The addition of azoles with chlorhexidine may enhance its effectiveness for resilient bacterial infections. Ketoconazole with chlorhexidine enhances its effectiveness when challenged with yeast infections. However, miconazole and chlorhexidine may have an antagonistic effect against *Malassezia*, which should be further investigated. The possible benefit of adding azoles to chlorhexidine to limit antimicrobial use should also be weighed against the increasing threat of azole resistance being seen in *Malassezia* isolates. Broth microdilution studies using topical treatments can help us to understand topical efficacy and interactions in different pathogens. Finally, it is also important to note that although a statistically significant difference was seen in this *in vitro* study, the magnitude of difference was one double dilution. Although this may seem potentially associated with a minimal clinical benefit, these results may suggest that chlorhexidine may still be effective against the organisms tested even in cases in which shampoos are overdiluted. Future studies should investigate the effects of topical chlorhexidine–azole products *in vivo* and *in vitro* using other bacterial and fungal causes of skin infections.

## Supplementary material

10.1099/mic.0.001701 Uncited Supplementary Material 1.

## Abbreviations

ATCC: American Type Culture Collection
MBC/MFC: minimum bactericidal or fungicidal concentration
*MDR-SP*: multidrug-resistant *SP*
MHB: Mueller–Hinton broth
*MP*: *Malassezia pachydermatis*
PEG400: polyethylene glycol 400
*SP*: *Staphylococcus pseudintermedius*
