# Supplementary material for: In vitro evaluation of the antimicrobial activity of chlorhexidine alone or in combination with ketoconazole or miconazole against clinical isolates of Malassezia pachydermatis and multidrug-resistant Staphylococcus pseudintermedius
Source: Microbiology (Reading). 2026 Apr 28;172(4):001701. doi: 10.1099/mic.0.001701 (PMC13128280; doi:10.1099/mic.0.001701)
Supplement: Uncited Supplementary Material 1. [file mic-172-01701-s001.pdf]

Table S1: Minimum Inhibitory Concentration (MIC), Minimum Bactericidal Concnetration (MBC), and Minimum Fungicidal Concentration (MFC) data for chlorhexidine, miconazole, and ketoconazole and respective combinations (Chlor/Mico and Chlor/Keto) in ATCC strains of *Staphylococcus pseudintermedius* and *Malassezia pachydermatis.*

		**Chlorhexidine (µg/mL)**	**Miconazole (µg/mL)**	**Ketoconazole (µg/mL)**	**Chlor/Mico (µg/mL)**	**Chlor/Keto (µg/mL)**
Staphylococcus pseudintermedius (ATCC 49444)	Median MIC	1.5	2.0	ND	1.5/1.0	0.75/10
	Median MBC	6.0	ND	ND	3.0/2.0	3.0/40
Malassezia pachydermatis (ATCC 14522)	Median MIC	2.0	0.16	0.08	1.0/0.08	1.0/0.04
	Median MFC	2.0	0.16	0.08	2.0/0.16.0	1.0/0.04

Table S2: Effects of the azole/chlorhexidine combinations versus chlorhexidine alone (Δ) against *Staphylococcus pseudintermedius* isolates (n=30). Synergy (S): reduction in 2 log_{10} colony forming units (CFU)/mL; Additive/Indifference (A/I): reduction in 1 log_{10} CFU/mL; Antagonism (A): increase in 2 log_{10} CFU/mL

	Log10 CFU/mL			Δ with chlorhexidine		Interpretation	
**Isolate #**	Chlorhexidine	Chlor/Mico	Chlor/Keto	Chlor/Mico	Chlor/Keto	Chlor/Mico	Chlor/Keto
1	9.66	8.42	10.25	1.24	-0.59	A/I	
2	9.29	8.35	8.95	0.94	0.34		
3	9.28	8.05	9.01	1.23	0.27	A/I	
4	9.37	8.27	8.99	1.1	0.38	A/I	
5	9.84	9.26	10.02	0.58	-0.18		
6	9.62	8.72	9.81	0.9	-0.19		
7	8.53	1	10.07	7.53	-1.54	S	
8	9.97	9.02	9.4	0.95	0.57		
9	9.94	9.19	10.19	0.75	-0.25		
10	9.57	8.92	9.47	0.65	0.1		
11	9.41	8.69	9.41	0.72	0		
12	8.78	8.88	10.09	-0.1	-1.31		
13	9.48	8.8	10.1	0.68	-0.62		
14	8.48	8.05	8.35	0.43	0.13		
15	9.26	8.65	9.49	0.61	-0.23		
16	9.13	8.83	9.26	0.3	-0.13		
17	8.97	1	9.36	7.97	-0.39	S	
18	9.15	9.32	9.79	-0.17	-0.64		
19	9.62	9.29	9.87	0.33	-0.25		
20	9.43	7.88	9.19	1.55	0.24	A/I	
21	8.92	7.88	8.94	1.04	-0.02	A/I	
22	9.29	1	8.42	8.29	0.87	S	
23	9.57	8.42	9.45	1.15	0.12	A/I	
24	9.2	8.35	9.36	0.85	-0.16		
25	8.78	8.05	8.8	0.73	-0.02		
26	8.18	8.78	8.78	-0.6	-0.6		
27	9.39	9.5	10.06	-0.11	-0.67		
28	10.01	8.05	7.86	1.96	2.15	A/I	S
29	9.3	8.27	9.32	1.03	-0.02	A/I	
30	8.75	8.35	10.19	0.4	-1.44		

Table S3: Effects of the azole/chlorhexidine combinations versus chlorhexidine alone (Δ) against *Malassezia pachydermatis* isolates (n=30). Synergy (S): reduction in 2 log_{10} colony forming units (CFU)/mL; Additive/Indifference (A/I): reduction in 1 log_{10} CFU/mL; Antagonism (A): increase in 2 log_{10} CFU/mL

	Log10 CFU/mL			Δ with chlorhexidine		Interpretation	
**Isolate #**	Chlorhexidine	Chlor/Mico	Chlor/Keto	Chlor/Mico	Chlor/Keto	Chlor/Mico	Chlor/Keto
3	7.99	7.3	1	0.69	6.99		S
5	1	11.7	10.9	-10.7	-9.9	A	A
8	11.65	11.89	1	-0.24	10.65		S
10	11.63	10.9	1	0.73	10.63		S
11	10.18	9.9	1	0.28	9.18		S
12	10.84	10.3	1	0.54	9.84		S
13	7.7	11.29	1	-3.59	6.7	A	S
14	1	9.92	1	-8.92	0	A	
15	1	10.18	1	-9.18	0	A	
16	9.4	10.7	1	-1.3	8.4		S
17	1	10	9.88	-9	-8.88	A	A
18	1	8.48	1	-7.48	0	A	
19	11.6	9.88	1	1.72	10.6	A/I	S
20	1	11.9	1	-10.9	0	A	
21	1	10.88	1	-9.88	0	A	
22	9.66	11.14	1	-1.48	8.66		S
23	7.67	9.11	1	-1.44	6.67		S
24	10.47	11	1	-0.53	9.47		S
25	8.6	8.7	1	-0.1	7.6		S
26	7.18	10.2	1	-3.02	6.18	A	S
27	11.78	11.16	8.24	0.62	3.54		S
28	11.58	11.82	9.14	-0.24	2.44		S
29	9.57	12	10.17	-2.43	-0.6	A	
30	9.9	12	1	-2.1	8.9	A	S
31	11.27	12.17	1	-0.9	10.27		S
32	9.94	12	1	-2.06	8.94	A	S
34	10.36	11.7	9.9	-1.34	0.46		
35	1	12	12.14	-11	-11.14	A	A
36	8.89	12.07	1	-3.18	7.89	A	S
37	1	10.18	10.89	-9.18	-9.89	A	A
